# TOPAS Simulation of the Mevion S250 compact proton therapy unit

**DOI:** 10.1002/acm2.12077

**Published:** 2017-04-26

**Authors:** Michael Prusator, Salahuddin Ahmad, Yong Chen

**Affiliations:** ^1^ Department of Radiation Oncology Stephenson Oklahoma Cancer Center University of Oklahoma Health Sciences Center Oklahoma City OK USA

**Keywords:** mevion, monte carlo, proton, TOPAS

## Abstract

As proton therapy becomes increasingly popular, so does the need for Monte Carlo simulation studies involving accurate beam line modeling of proton treatment units. In this study, the 24 beam configurations of the Mevion S250 proton therapy system installed recently at our institution were modeled using the TOolkit for PArticle Simulation (TOPAS) code. Pristine Bragg peak, spread out Bragg peak (SOBP), and lateral beam profile dose distributions were simulated and matched to the measurements taken during commissioning of the unit. Differences in the range for all Percent Depth Dose (PDD) curves between measured and simulated data agreed to within 0.1 cm. For SOBP scans, the SOBP widths all agreed to within 0.3 cm. With regards to lateral beam profile comparisons between the measured and simulated data, the penumbras differed by less than 1 mm and the flatness differed by less than 1% in nearly all cases. This study shows that Monte Carlo simulation studies involving the Mevion S250 proton therapy unit can be a viable tool in commissioning and verification of the proton treatment planning system.

## 1 Introduction

Success in radiation therapy is dependent on maximization of the tumor control probability while minimizing the normal tissue complication probability. The characteristics of the proton Bragg peak help to deliver large doses to the target, lower entrance doses proximal to the target and almost no doses distal to the target.^1^ Advancements in Monte Carlo simulation and radiation transport calculations have enabled more accurate characterizations of the radiation fields during proton treatments that can benefit patients.^2^ The cases where the greatest advantages of proton therapy could be realized are in targets near critical organs and treatments involving pediatric patients.^3^, ^4^, ^5^

In order for the full potential of proton therapy to be utilized, there is a need for an accurate dose calculation in proton treatment plans. Monte Carlo simulation has traditionally been shown as a prominent method for conducting various research topics that include dosimetric, linear energy transfer (LET), and commissioning studies.^6^, ^7^, ^8^ In a study done by Paganetti et al., it was shown that the modeling of the IBA proton treatment head at the Northeast Proton Therapy Center resulted in simulated data matching with measured beam data within millimeter accuracy for beam range and 3 mm for SOBP width.^9^ This acceptable tolerance helps generation of detailed simulated beam data for use in commissioning of a treatment planning system.^10^ Monte Carlo simulation has also been used to calculate the risk of secondary cancer due to neutron exposure occurring from nuclear reactions in the treatment system on patients that have undergone proton craniospinal irradiation treatments.^11^ Paganetti has also reported that Monte Carlo simulations can even improve proton beam range uncertainty by up to 2.2%.^12^

A passive double scattering compact proton therapy unit has recently been installed at our facility. The Mevion S250 is the first proton therapy system of its kind, delivering a pulsed beam with a nominal energy of 250 MeV and utilizing an in‐room superconducting synchro‐cyclotron mounted on a gantry that allows for 180° rotation. The compact nature of the unit has many cost and therapy treatment advantages, and eliminates the need for a complex beam transport system.^13^ The main beam shaping components in the nozzle of the unit include a lead first scattering foil present at the cyclotron exit for initial beam spread. The beam then passes through a bimaterial staircase type range modulator wheel (RMW) consisting of one track made of graphite to modulate the range of each Bragg peak, and a second track made of lead to ensure uniform scattering power over all steps of the wheel. The last step of each wheel has a brass wedge to completely stop the beam. A bilayer contoured second scattering foil made from lead and Lexan is incorporated downstream of the RMW to further spread and flatten the beam. The last components the beam passes through are a graphite absorber for energy degradation and a post absorber for fine tuning the beam range, followed by two ion chambers to monitor beam output. For more information on the initial clinical experience with the system, the reader is referred to Zhao et al.^13^

**Figure 1:**
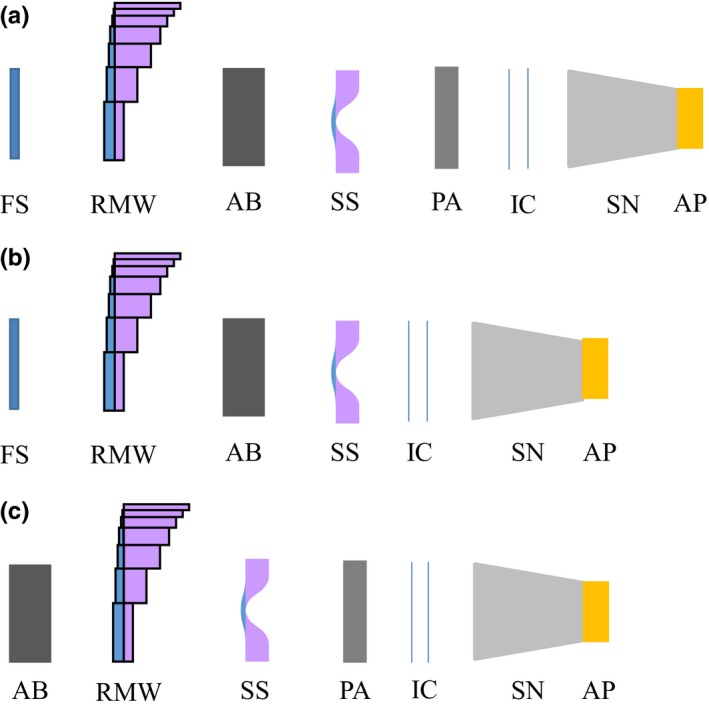
The configurations of beam line components for (a) the large group of configurations, (b) the deep group of configurations and (c) the small group of configurations. Where FS is the first Scattering foil, RMW is the range modulator wheel, AB is an energy degrader, SS is the second scattering foil, PA is the post absorber, IC are the ion chambers, SN is the snout and AP is the aperture.

The Mevion S250 has 24 different beam configurations, divided into large, deep, and small groups. The large group utilizes a large beam nozzle with an uncollimated field size of 25 cm in diameter where the deep and small group share a small beam nozzle with the field size of 14 cm in diameter. The distinction between deep and small groups occurs in their range capabilities and modulation widths. The deep group has a depth range from 20.1–32 cm with a maximum modulation width of 10 cm, whereas the small group has a shallower depth range of 5–20 cm but has a maximum modulation width of 20 cm. For any given group, a special beam configuration results from the unique order and combination of different beam line components as summarized in Fig. 1. In total, the Mevion system offers 12 beam configurations in the large group, 5 for the deep group and 7 in the small group which are composed from 18 first scattering foils, 14 RMWs, and 3 secondary scattering foils. Table 1 shows the beam characteristics for all configurations in each group.

**Table 1:** Summarization of the three beam configuration groups used in the Mevion S250

Group	Number of configurations	Max. range (cm)	Min. range (cm)	Max. SOBP width (cm)	Min. SOBP width (cm)
Large	12	25	5	20	2
Deep	5	32	20.1	10	2
Small	7	20	5	20	2

The objective of the this study has been to model the beam line components for all 24 beam configurations of the Mevion S250 system using Monte Carlo simulation in order to accurately characterize the radiation field exiting the treatment head, and benchmark the simulation results with the measured beam data obtained during commissioning of the machine.

## 2 Methods

During the commissioning of the Mevion system, pristine Bragg peaks and full SOBP scans were measured with PTW's advanced Markus ion chamber (PTW 34045) in IBA's Big Blue 1 water phantom. The chamber is a parallel plate ion chamber with a sensitive volume of 0.02 cubic centimeters, enabling the detector to accurately measure the sharp distal falloff in the proton PDD. The lateral beam profile scans were measured in the same phantom using PTW's proton diode detector (PTW 60020) with a sensitive volume of 0.03 cubic millimeters. The diode detector provided the necessary spatial resolution needed for capturing the sharp penumbra seen in lateral beam profile scans. This specific diode was chosen in part due to its resilience against radiation damage. McAuley et al. showed that a loss of sensitivity as a function of accumulated dose was only 1% per 100 Gy, which is well under the doses delivered during our measurements.^14^

TOPAS (TOolkit for PArticle Simulation) version 2.0 was utilized to model the beam delivery system in this study.^15^ TOPAS 2.0 is an extension of the GEANT4 10.1.p02. toolkits that was developed specifically as a user friendly proton therapy tool. It has been validated experimentally as a viable choice when tasked with reproducing beam data from a passive scattering proton system.^16^ The dimensions and materials for each beam component were modeled in TOPAS based on the data given by the manufacturer. For all simulations, the TOPAS default modular physics list was used, which is composed of “TsEmStandardPhysics_option3_WVI”, “HadronPhysicsQGSP_BIC_HP”, “G4DecayPhysics”, “G4IonBinaryCascadePhysics”, “G4HadronElasticPhysicsHP”, “G4StoppingPhysics”, and “G4RadioactiveDecayPhysics”. The range cut for secondary particles was set to 0.005 cm, with an energy cut in water of 990 eV, 57.3 keV, 5 keV, and 56.6 keV for gamma rays, electrons, protons, and positrons respectively. A water phantom with dimensions of 40 × 40 × 40 cm^3^, placed downstream of the delivery nozzle, was used.

### 2.A Pristine bragg peak simulations

For all configurations, the deepest pristine Bragg peak simulations were calculated by passing beam through the full beam shaping geometry including a static RMW with only the thinnest step in the beam path. An open ring aperture was located at 180 cm downstream of the source. The diameter of the aperture was 14 cm for the small and deep groups and 25 cm for the large group. The airgap between the bottom face of the aperture to the top surface of the water was set to 10 cm. The detector mesh used to score the dose was 2 × 2 × 0.1 cm. The face of the detector mesh was large enough to allow for fewer particles to be run achieving a smooth PDD, while the fine resolution in the z direction prevented distortion of high gradient areas.

### 2.B Spread out bragg peak simulations

The Mevion proton system uses a pulsed beam through a RMW rotating at a constant speed of 600 rpm to deliver protons to the target. A user defined beam current modulation (BCM) sequence is applied to synchronize and scale the individual beam pulse striking on the rotating RMW to achieve a uniform, flat SOBP. To mimic such process, individual pristine Bragg peaks were created in simulation in a manner similar to Polf et al.^17^ by determining the timing and landing locations of the pulses on each step of the wheel. The pulses were weighted so that each step the beam passed through received the same number of protons. For our purposes, each step received 3 × 10^6^ protons, regardless of the number of pulses located on the step.

The pristine Bragg peaks from each step of the wheel were scaled by applying weighting factors to create a SOBP that matched to the measured data (see Eq. (1)).

(1) $$D\left(d\right)=\sum_{i}^{j}w_{i}p{\left(d\right)}_{i}$$

where $D\left(d\right)$ is the dose as a function of depth, $w_{i}$ is the weighting factor applied to the *i*th peak, and $p{\left(d\right)}_{i}$ is the dose distribution associated with the *i*th pristine Bragg peak.

The sum of square errors between measured and simulated data for each point at 0.1 cm depth increments were minimized to give the best agreement between the calculated SOBP and commissioning data (see Eq. 2).

(2) $$SSE=\sum_{d_{1}}^{d_{2}}{\left(D{\left(d\right)}_{m}-D{\left(d\right)}_{s}\right)}^{2}$$

where SSE is the sum of square error, $D{\left(d\right)}_{m}$ is the dose distribution for the measured data and $D{\left(d\right)}_{s}$ is the dose distribution for the simulated data.

**Figure 2:**
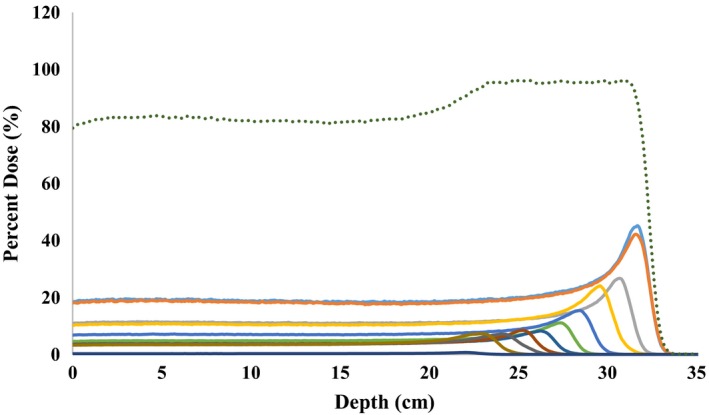
Individual peaks created from the method described in the text to be summed to create the SOBP. Each peak was assigned a specific weighting factor that, when summed together with the other peaks, created a flat SOBP shown by the dotted green curve.

An example of an SOBP and corresponding pristine Bragg peaks for configuration 13 are shown in Fig. 2. Three SOBPs (one configuration from each of the group categories) were constructed using this method (detailed information is shown in Table 2).

**Table 2:** Range and modulation widths of the three SOBP beams chosen for the simulation

Configuration	Range (cm)	Modulation width (cm)	Air gap (cm)	Aperture size (cm)
Large	25	20	10	18 × 18
Deep	32	10	10	8 × 8
Small	15.3	15.3	10	8 × 8

### 2.C Lateral beam profile matching

In a passive scattered proton system, scattering foils are put in place to spread the beam to a clinically relevant treatment size. For Mevion S250, the small and deep groups support a maximum field size of 14 × 14 cm^2^, while the large group supports field sizes up to 25 × 25 cm^2^. The spreading of the beam is achieved through the use of two scattering foils (a first scatter foil and a second scatter foil). The second scatter foil is a bi‐material foil fabricated from lead and Lexan. The lead functions to further spread and flatten the beam while the Lexan is added to create a constant water equivalent thickness (WET) across the component. Three unique second scatter foils are equipped in the system (one for each group). To ensure that the scattering foils were modeled accurately, lateral profiles were simulated for two different configurations within each group. For each of these beam configurations, lateral beam profiles were calculated at a shallow and deep depth. The detector mesh was 0.1 × 2 × 2 cm. The base of the aperture was placed 10 cm above the water surface, and isocenter was located at a depth of the range minus half the modulation width of the configuration being simulated. For each simulation, 2.5 × 10^8^ protons were run. Table 3 gives a summary of each of the configurations and depths used to calculate the lateral beam profile.

**Table 3:** Range and depths of the three configurations chosen for lateral beam profile calculation

Group	Range (cm)	Shallow depth (cm)	Deep depth (cm)	Aperture size (cm)
Large	25	10	20	18 × 18
Large	16.7	5	10	18 × 18
Deep	32	10	20	8 × 8
Deep	22	10	15	8 × 8
Small	20	10	15	8 × 8
Small	15	5	10	8 × 8

### 2.D Spread out bragg peak modulation width adjustments

In the clinical applications of a passive scatter system, it is necessary to adjust the modulation size of the SOBP for target coverage. The Mevion system offers the adjustment for the SOBP width in 0.1 cm increments by applying stop digits in the BCM files. These stop digits designate a stop pulse, which reduces or cuts the beam current on a certain pulse of the incident beam. In these cases, a step of the wheel may receive less fluence of protons or none at all. By eliminating or lowering the fluence contribution to the shallower peaks, the modulation width will become smaller. Using a similar idea, adjustment of the modulation width in our simulation can be done through simple scaling of weighting factors for the individual pristine Bragg peaks. For each configuration, we sequentially subtracted one pulse from our fully modulated beam and calculated the new SOBP widths. This gave a curve of SOBP width vs. stop pulse that could easily be looked up to determine what the new weighting factors were for each peak to create the desired modulation width.

## 3 Results and discussion

### 3.A Pristine bragg peaks

**Figure 3:**
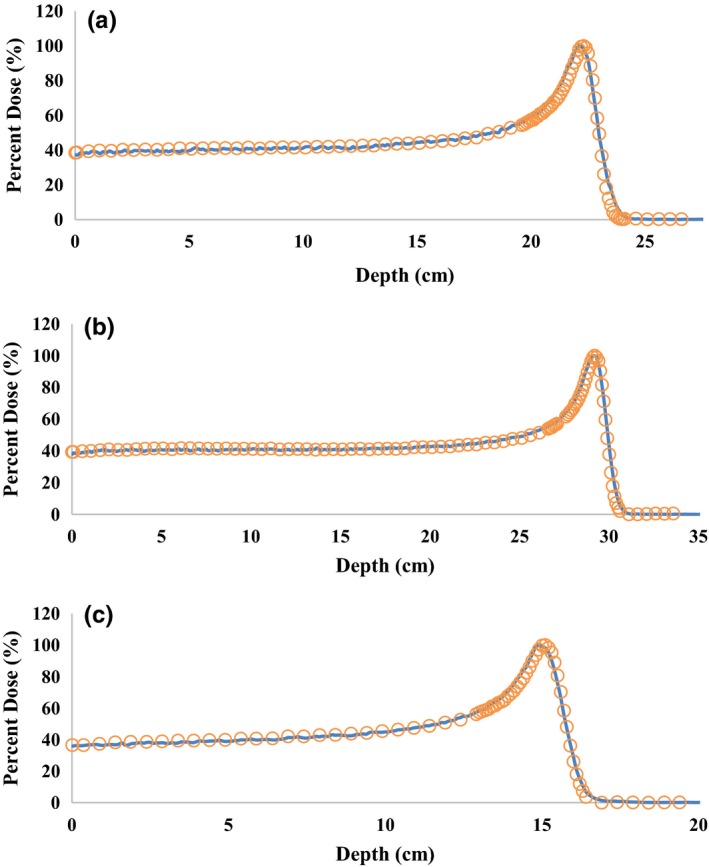
Measured data from commissioning (circles) and simulated (solid lines) normalized pristine Bragg peaks from (a) a large configuration with a range of 22.5 cm, (b) deep configuration with a range of 29.5 cm and (c) a small configuration with a range of 15.3 cm.

The simulated deepest Pristine Bragg peaks for each configuration were normalized and compared with the corresponding measured data at PDD of 0.5 cm (PDD (0.5)) and the range of the beam, defined as the depth of 90% dose (D90). For all 24 beam configurations, the range matched to within millimeter accuracy and the PDD (0.5) was within 2%. The average differences over the large group for the PDD (0.5) and D90 depth was 0.9% (ranged from 0.4 to 1.5%) and 0.06 cm (ranged from 0.04 to 0.09 cm), respectively (shown in Table 4). The average differences over the deep group for the PDD (0.5) and D90 depth was 1.3% (ranged from 0.6 to 1.8%) and 0.03 cm (ranged from 0 to 0.04 cm) respectively (shown in Table 5). The average differences over the small group for the PDD (0.5) and D90 depth was 1.2% (ranged from 0.3 to 1.9%) and 0.04 cm (ranged from 0.00 to 0.07 cm) respectively (shown in Table 6). An example from each group is shown in Fig. 3, where normalized depth dose curves from beam commissioning measurements and simulated data are plotted for comparison.

**Table 4:** Absolute Differences between measured and simulated percent depth dose data for the large group

Configuration	PDD (0.5) difference (%)	Measured D90 (cm)	Simulated D90 (cm)	Difference in D90 (cm)
1	0.8	25.10	25.06	0.04
2	0.8	22.59	22.54	0.05
3	0.4	20.92	20.87	−0.05
4	0.5	18.78	18.71	0.07
5	1.1	16.80	16.74	0.06
6	1.5	14.94	14.90	0.04
7	1.0	13.24	13.19	0.05
8	0.6	11.52	11.61	−0.09
9	1.2	10.07	10.02	0.05
10	0.5	8.72	8.63	0.09
11	1.5	7.39	7.31	0.08
12	1.3	6.67	6.58	0.09
Avg.	0.9	–	–	0.06

**Table 5:** Absolute Differences between measured and simulated percent depth dose data for the deep group

Configuration	PDD (0.5) difference (%)	Measured D90 (cm)	Simulated D90 (cm)	Difference in D90 (cm)
13	1.2	31.88	31.84	0.04
14	0.6	29.51	29.51	0.00
15	1.6	27.08	27.06	0.02
16	1.8	24.55	24.52	0.03
17	1.1	22.06	22.02	0.04
Avg.	1.3	–	–	0.03

**Table 6:** Absolute Differences between measured and simulated percent depth dose data for the small group

Configuration	PDD (0.5) difference (%)	Measured D90 (cm)	Simulated D90 (cm)	Difference in D90 (cm)
18	1.5	20.05	20.00	0.05
19	1.9	17.81	17.74	0.07
20	0.3	15.39	15.32	0.07
21	1.5	13.32	13.30	0.02
22	1.7	11.28	11.24	0.04
23	0.6	9.18	9.18	0.00
24	1.2	7.12	7.12	0.00
Avg.	1.2	–	–	0.04

To achieve agreement between the measured data and the simulated data for the pristine Bragg peak of each configuration, small adjustments were made to the manufacturer provided geometry of the beam delivery system. As described by Bednarz et al.,^18^ there is uncertainty as to how accurate the provided blueprints reflect the real geometry of the components in the commissioned treatment head. Therefore, tuning some of the physical parameters of the geometry was necessary assuming that the adjustments were within manufacturer tolerance.^19^ The range of proton beam was first matched by fine adjustments of the post absorber thicknesses using stopping power conversion ratios. In most cases, after range correction, the entrance dose and Bragg peak distal fall off needed to be tuned. Paganetti and Bednarz showed that the beam spot size, energy, and angular spread can influence the shape of the Bragg curve and provides one of the largest sources of uncertainty, due to the difficulty in measuring these parameters.^10^, ^18^ Paganetti et al., also showed that beam energy spread has the largest influence on entrance dose and distal fall off.^10^ The same trend is also found in our study, where increasing the energy spread increased the entrance dose and the distal fall off length. Therefore, in order to tune our pristine Bragg peaks to appropriate distal falloff, the energy spread was adjusted iteratively until the best agreement between measured and simulated data was reached. We found that the energy spread that achieved the best agreement between the measured and the simulated data for most of the configurations was 0.4%. The angular spread and spot size were then optimized in order to achieve further agreement in data. A 0.005 radian standard deviation in angular spread in both the x and y directions gave the best representation of commissioned data, along with a beam spot size of 8 mm for the majority of configurations.

In the case of the large configuration group, some of the shallower range configurations were not able to be tuned to match within our set criteria with adjustments to the source alone. The Mevion system has a consistent distal falloff margin regardless of the energy or configuration. We found that as we decreased the energy of the beam and increased the field size, the distal fall off margins in simulation began to degrade. This is likely due to the presence of more material in the beam that introduced large uncertainty in geometry; and influenced the beam energy distribution in a way not accounted for in our simulations. In these specific cases, small adjustments (on the order of a tenth of millimeter) to the thickness of the first scattering foil were made in order to adjust the distal gradient to agree with measurements. These adjusted first scattering foils were used in the geometry for the subsequent Bragg peak simulations.

### 3.B Spread out bragg peak matching

**Figure 4:**
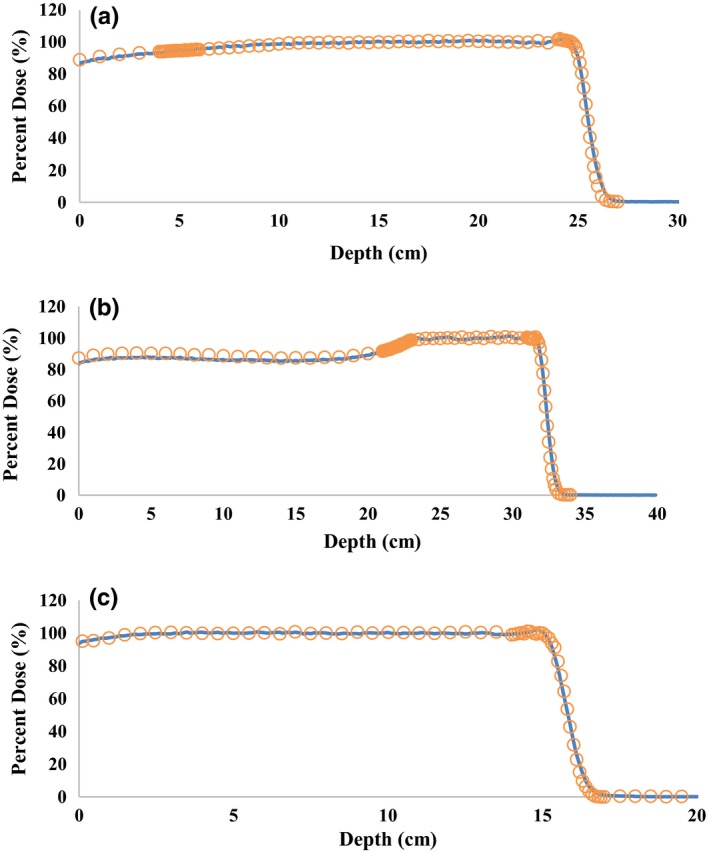
Measured data from commissioning (circles) and simulated (solid lines) normalized spread out Bragg peaks for (a) large configuration (configuration 1) with a range of 25 cm, (b) deep configuration (configuration13) with a range of 32 cm, and a (c) small configuration (configuration 20) with a range of 15.3 cm.

At our institution, SOBP width is defined as the distance between the proximal 95% dose depth and the distal 90% dose depth. To evaluate the agreement between simulated results and measured data, the SOBP width, beam range, and the depth of distal 20% dose were compared. Figure 4 shows the matching of normalized percent depth dose curves for configuration 1, 13, and 20. For all three SOBPs, the distal 90% depths were matched within 0.1 cm difference. The differences of distal 20% depths were within 0.15 cm and the largest discrepancies in SOBP width was 0.24 cm for configuration 1 as shown in Table 7. This has been a difficult characteristic to precisely reproduce SOBP width in simulation due to the nature of shallow gradient at the proximal end of the PDD curve. The main contribution to the proximal end of a SOBP is from the beam pulses hitting on the finite steps of the RMW. It has been shown as partial shining effect that even small temporal errors in the BCM and RMW synchronization can result in substantial errors for SOBP formation.^19^ Furthermore, studies have been done on optimization of the BCM in Monte Carlo simulation for a continuous beam from an isochronous cyclotron, but there has been little work done on how to optimize this for a pulsed beam from a synchrocyclotron.^10^, ^19^ Nevertheless, benchmarking simulated SOBP width within 0.3 cm to measurement data are in tolerance when commissioning a Monte Carlo simulation model for dose calculation.^9^ This agreement was achieved by actually determining the step location for each beam pulse which is necessary to represent well in SOBP simulations due to the presence of peak broadening that occurs when a pulse strikes the edge of a step.

**Table 7:** Comparison of measured and simulated SOBP for configurations 1, 13, and 20

Configuration	Measured modulation width (cm)	Simulated modulation width (cm)	Mod. width difference (cm)	D90 difference (cm)	D20 difference (cm)
Large	19.78	20.02	−0.24	0.04	0.14
Deep	9.77	9.93	−0.16	0.07	0.10
Small	15.41	15.33	0.08	0.08	0.07

### 3.C Lateral beam profile matching

**Figure 5:**
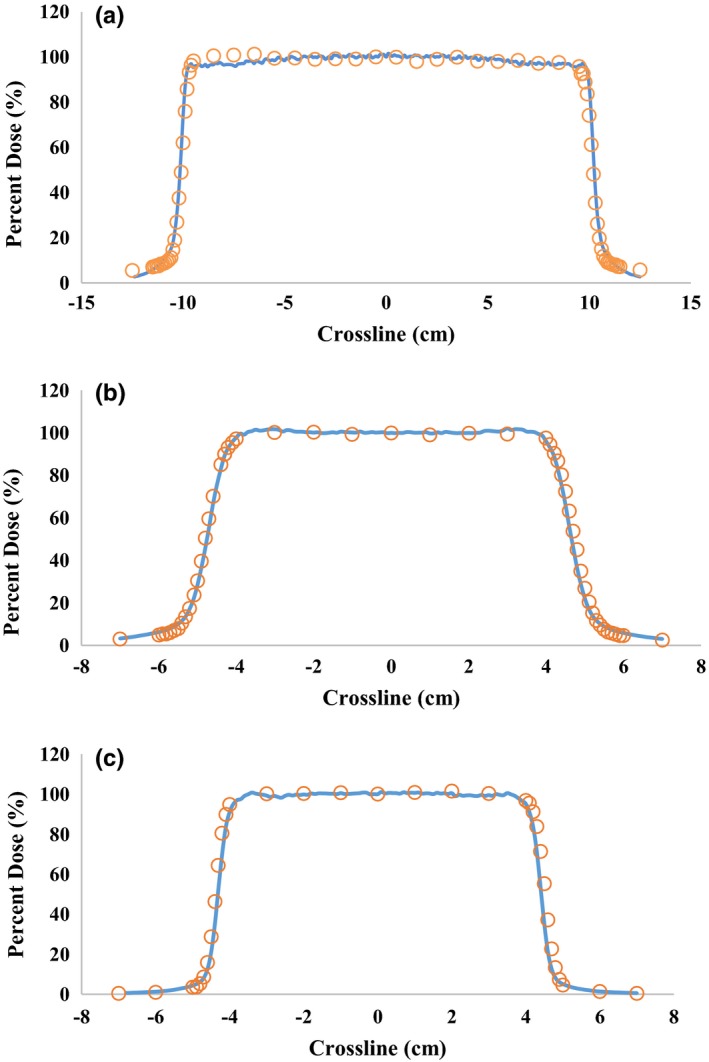
Measured data from commissioning (circles) and simulated (solid lines) normalized lateral beam profiles from a (a) large configuration (configuration 1) at a depth of 10 cm, (b) deep configuration (configuration 13) at a depth of 20 cm, and (c) small configuration (configuration 20) at a depth of 10 cm.

Three of the lateral beam profiles are shown in Fig. 5 with the simulated and measured data plotted on each graph. The penumbras were calculated as the distance between the 80% and 20% dose levels. Beam flatness and symmetry was calculated using Equations 3 and 4 respectively.

(3) $$F=\frac{D_{min}-D_{max}}{D_{min}+D_{max}}*100$$

where $D_{min}$ and $D_{max}$ are the minimum and maximum doses within the middle 80% of the field size.

(4) $$S=\frac{DL-DR}{DL+DR}*100$$

where DL and DR are the integral doses of the left and right side of the radiation field respectively.

The full width at half maximum values (FWHM) for both measurement and simulated data were calculated and compared with each other. The absolute differences in the penumbras between simulated and measured profiles at each depth for each configuration all agreed to well within a millimeter. Flatness and Symmetry values for all 12 profiles (six configurations at two depths each) are listed in Table 8. All of the full width at half maximum (FWHM) calculations agreed to within 0.2 cm. It has been noticed that subtle changes in the physical dimensions of the aperture cut‐out or air gap setting could affect in large the simulated field size due to the diverging nature of the beam.

**Table 8:** Comparison of measured and simulated cross‐profiles for two configurations in each group

Config./Group	Depth (cm)	Penumbra difference (cm)	Simulated flatness (%)	Measured flatness (%)	Simulated symmetry (%)	Measured symmetry (%)	FWHM difference (cm)
1 (Large)	10	0.03	2.9	2.0	0.6	1.9	0.12
	20	0.06	2.6	2.2	0.3	0.9	0.02
5 (Large)	5	0.06	2.7	1.9	0.1	0.9	0.01
	10	0.04	2.3	1.5	0.6	0.5	0.00
13 (Deep)	10	0.01	1.9	1.0	0.1	0.1	0.11
	20	0.01	1.2	0.6	0.3	0.2	0.13
17 (Deep)	10	0.01	1.0	2.2	0.5	1.1	0.16
	15	0.02	1.8	1.4	0.3	1.5	0.18
18 (Small)	10	0.02	1.1	1.5	0.1	1.2	0.02
	15	0.02	1.3	1.4	0.2	0.5	0.01
20 (Small)	5	0.03	1.2	1.6	0.6	0.4	0.15
	10	0.03	1.5	0.8	0.1	0.1	0.17

### 3.D Spread out bragg peak modulation width adjustments

**Figure 6:**
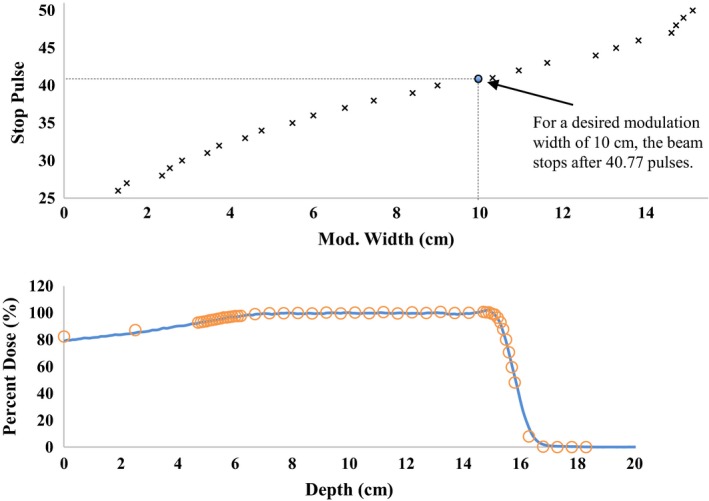
(a) The stop pulse curve for configuration 21. The curve will give the appropriate stop pulse to use when adjusting the SOBP width for simulations. Linear interpolation is used to determine the fraction of the last stop pulse to be applied when the desired modulation width is between pulses. This curve was used to provide the stop pulse to simulate the reference beam at our institution. (b) The measured data from commissioning (circles) and simulated (solid lines) normalized spread out Bragg peak for the reference SOBP.

Based on the timing of beam pulse and the speed of the rotating RMW, there are roughly 50 contributing pulses per full rotation of the modulator wheel. The intensity of each of the pulses is also modulated to achieve the preset SOBP width with 0.1 cm accuracy. Using the modulation adjustment algorithm described in the methods section, a reference SOBP with various modulation widths were simulated and shown together with a pulse modulation function in Fig. 6. If the intended modulation width does not correspond to an integer stop pulse, one can use linear interpolation to determine the fraction of the last pulse to be applied to the corresponding pristine Bragg peak. In this example, the desired SOBP width was changed to 10 cm, resulting in 40.77 pulses per revolution of the modulator wheel.

The measured modulation width was 10.1 cm and the simulated modulation width was 9.94 cm, giving a difference of only 0.17 cm. The depth at 90% dose differed between the two curves by 0.02 cm. The depth of 20% dose shows an absolute difference of 0.07 cm between simulated and measured beam data. Our method of SOBP width adjustment thus shows to be a reasonable option when simulating a pulsed proton beam from a synchrocyclotron.

## 4 Conclusion

To our knowledge, this study marks the first full scale Monte Carlo simulation of the Mevion double scattering S250 proton therapy system. The beam line components for all 24 beam configurations were extensively modeled and we have shown excellent agreements for the simulated range and modulation width of given SOBP with the corresponding measured beam data taken during commissioning. This demonstrates that the simulation could serve as a promising tool for generating commissioning data for treatment planning system with acceptable accuracy and possible reduction of the intensive time required in measured data collection for the commissioning and verification of the proton treatment planning system.

## Conflicts of interest

The authors have no relevant conflicts of interest to disclose.
